# Influence of Physical Activity Interventions on Body Representation: A Systematic Review

**DOI:** 10.3389/fpsyt.2020.00099

**Published:** 2020-03-19

**Authors:** Duangkamol Srismith, Leona-Magdelena Wider, Hong Yu Wong, Stephan Zipfel, Ansgar Thiel, Katrin Elisabeth Giel, Simone Claire Behrens

**Affiliations:** ^1^Medical University Hospital Tübingen, Department of Psychosomatic Medicine and Psychotherapy, Tübingen, Germany; ^2^Max Planck Institute for Intelligent Systems, Department of Perceiving Systems, Tübingen, Germany; ^3^Graduate Training Centre of Neuroscience, International Max Planck Research School, University of Tübingen, Tübingen, Germany; ^4^Werner Reichardt Centre for Integrative Neurosciences, University of Tübingen, Tübingen, Germany; ^5^Department of Philosophy, University of Tübingen, Tübingen, Germany; ^6^Institute of Sports Science, Faculty of Economic and Social Sciences, University of Tübingen, Tübingen, Germany

**Keywords:** body image, body representation, physical activity, eating disorders, body dysmorphic disorders

## Abstract

Distorted representation of one's own body is a diagnostic criterion and corepsychopathology of disorders such as anorexia nervosa and body dysmorphic disorder. Previousliterature has raised the possibility of utilising physical activity intervention (PI) as atreatment option for individuals suffering from poor body satisfaction, which is traditionallyregarded as a systematic distortion in “body image.” In this systematic review,conducted according to the PRISMA statement, the evidence on effectiveness of PI on body representation outcomes is synthesised. We provide an update of 34 longitudinal studies evaluating the effectiveness of different types of PIs on body representation. No systematic risk of bias within or across studies were identified. The reviewed studies show that the implementation of structured PIs may be efficacious in increasing individuals’ satisfaction of their own body, and thus improving their subjective body image related assessments. However, there is no clear evidence regarding an additional or interactive effect of PI when implemented in conjunction with established treatments for clinical populations. We argue for theoretically sound, mechanism-oriented, multimethod approaches to future investigations on body image disturbance. Specifically, we highlight the need to consider expanding the theoretical framework for the investigation of body representation disturbances to include further body representations besides body image.

## Introduction

Disturbances in body representation have been identified as the crux of many debilitating psychiatric disorders, such as body dysmorphic disorder (1), body integrity identity disorder (2), somatoparaphrenia (3), and asomatognosia (4). It has also been proposed as a core psychopathology of eating disorders [e.g., (4–9)]—especially in anorexia and bulimia nervosa. Heightened body dissatisfaction has been interpreted as a predisposition indicator in subclinical populations. Individuals with body image concerns are reportedly more vulnerable to developing eating and dieting pathologies (10–13). As yet, however, mechanisms of change in body representation are still poorly understood, limiting mechanism-oriented interventions for prevention and treatment of disturbed body representation.

Previous literature has suggested that regular physical activity has beneficial effects on thephysical health of the body, as well as a significant impact on the level of satisfaction with whichthe body is perceived [e.g., (14–16)]. This is surprising, insofar as cross-sectional studies in individuals withhigh levels of physical activity suggest decreased rather than increased body satisfaction (17). However, it is important to note that there has been little critical appraisal of the existing studies. Previous systematic reviews and meta-analyses on the topic of physical activity and its potential interactions with body satisfaction have been conducted from an outcome-centric perspective [e.g., (14, 15, 18–20)]—focusing on evaluating the effectiveness of physical activity as an intervention for the improvement of individuals’ body satisfaction—with an emphasis on the affective and cognitive aspects (i.e., body image). Nevertheless, the question of which potential mechanisms might be responsible for the apparent shift in body image after the introduction of physical activity has not been inadequately addressed.

Despite the lack of a unified consensus on its exact nature, the term body image has been widelyused in research across psychology, neuroscience, and psychiatry. Body image can roughly becharacterised as the conscious, predominantly visual, mental representation of one’s own body,which in turn provides a basis upon which perceptual, cognitive, and affective attitudes toward thebody are assigned (21, 22). However, it is important to note that the current literature largely concurson the use of the term body image as a measure of body satisfaction—in that body image as anoutcome measure is interpreted as the degree with which individuals are satisfied with various aspects of themselves that is influenced by the visual aspect of their body (e.g., appraisal of body shape information). In this review, we therefore adopt the term body representation when referring to the broad range of mental representations of one’s own body, whereas body image only refers to cognitive-affective appraisal of the body. The fact that body image investigations in health research has so far been conducted from an almost exclusively perceptual-affective perspective is worth discussing.

Longo (23) argued that higher level representations of thebody are unlikely to emerge from abstract cognition alone. Rather, they are constructed through theinterplay of multiple distinct body representations. Not only do individuals have immediateknowledge of their body from within (i.e., bodily awareness through interoception), they are alsoable to objectively reflect on their own body from an external perspective, in the same way thatexternal objects are cognitively assessed (with regards to their shape, size, location, aesthetics, et cetera). Relying on neuroscientific evidence, Longo’s framework of body perception consists of multiple distinct body representations that are informed by different sensory modalities and can be arranged along two orthogonal axes: explicit vs. implicit & perceptual vs. conceptual. However, most empirical data concerning body image has been based on self-report or visual body size judgments, which effectively leads to the underrepresentation of other somatically driven/sensory inputs when considering the potential mechanism underlying the concept of body image (24). Although these subcomponents of body representation have been demonstrated to have distinct underpinning neural networks [see (25) for review], the mechanisms responsible for the development and regulation of these subcomponents are still very much unexplored (e.g., the idea that body satisfaction, or the lack thereof, could be socially and/or somatically driven—or the product of their interactions). As such, it is not at all clear why the body image should remain the sole focal point when investigating how individuals mentally represent their own body, and the potential distortions therein.

In this systematic review, we aim to synthesise existing literature investigating longitudinal interaction between physical activity and body image. Our purpose is to synthesise the empirical evidence from previous studies with a focus on effects, broader potential, and eventual impact mechanisms of PI on body representation. Specifically, our research questions were:

- Are there systematic effects of PI on body representation?
- Are previous studies informative with regard to prevention or treatment of sub-clinical or clinically relevant body image disturbance?
- Are there specific mechanisms of how long-term engagement in structured PI that influence thedynamics of individuals’ body representations?

## Method

The systematic review process was conducted according to the PRISMA statement (26). Methods of analysis and inclusion criteria were specified in advance and documented in a protocol.

### Literature Search

Studies were identified *via* searching the following electronic databases: PubMed, Web of Science and SPORTDiscus. The search was weekly updated until January 2020.

The specific search terms are as follows: (“body image” OR “body representation” OR “body dissatisfaction” OR “body satisfaction” OR “body image disturbance”) AND (“physical activity” OR “physical exercise” OR “exercise intervention” OR “endurance training” OR “exercise training” OR “exercise intervention” OR “aerobic exercise” OR “aerobic training” OR “anaerobic exercise” OR “anaerobic training” OR “motor activity” OR “resistance training” OR “resistance exercise” OR “strength training” OR “weight training” OR “weightlifting” OR “cardio training” OR “cardio” OR “athletic sports” OR “exercise program” OR “fitness training” OR “cardiovascular training” OR “interval training” OR “intermittent training” OR “interval exercise” OR “intermittent exercise” OR “sprint training” OR “sprint exercise” OR “high intensity interval training” OR “high intensity interval exercise” OR “moderate intensity training” OR “moderate intensity exercise” OR “exercise/psychology” OR “exercise therapy/methods” OR “resistance training/methods”).

Additionally, reference lists of included articles were hand searched.

### Eligibility Criteria and Study Selection

Each step of the eligibility assessment was performed independently by two reviewers according to the PICOS criteria (27). Only articles in English were considered.

Studies had to fulfil the following criteria:

*Population*: a sample of adult participants,
*Intervention*: include a longitudinal physical activity intervention (PI; no lifestyle counselling; specific targeting of body image/body representation not compulsory),
*Comparison/Control*: include a validated measure of body image/body representation, a control group was not required,
*Outcome*: at least one pre & post measurement of a validated self-report or experimental body image/body representation measure, and
*Study Type*: published as a peer-reviewed original article.

Exclusion criteria were as follows:

- samples younger than 18 years old, and
- no inference statistics performed.

The first author (DS) applied the search terms to three databases, extracted the search results and removed duplicates. DS and LW screened titles to identify relevant records. Abstracts and full texts of the identified records were subsequently screened by DS and SB. All studies included in the qualitative synthesis were rated according to the eligibility criteria by DS and SB. Interrater reliability between DS and LW for title screening was good (κ = 0.61; 94.2% agreement). Articles were included in the full-text screening if one reviewer rated it as a potential match. Interrater reliability between DS and SB for the subsequent steps was very good (κ = 1.0 for abstract screening; κ = 0.94 for full-text screening). Disagreements between the reviewers were solved by discussion and, when in doubt, articles were included. DS extracted relevant data from the included studies (i.e., sample characteristics, PI types and dosage, measure of fitness level, measures of body composition, measures of body image/body representation, and results).

### Risk of Bias in Individual Studies

To assess the risk of bias in individual studies, DS and SB conducted a quality rating. The Qualitative Assessment Tool for Quantitative Studies by the Effective Public Health Practice Project (28, 29) is recommended by the Cochrane Handbook for Systematic Reviews of Interventions to be used for assessing any quantitative study design (30) and was judged a suitable tool for systematic reviews of effectiveness (31). The tool consists of component ratings for the following categories: selection bias, study design, confounders, blinding, data collection methods, and withdrawals and dropouts. Components were rated according to the accompanying dictionary. Studies received ratings of either “strong”, “moderate”, or “weak” for each category. A global rating was assigned at the end of the process *via* the summarisation of the number of categories rated as “weak”. Disagreements between the reviewers were solved by discussion.

## Results

### Study Selection

**Figure 1:**
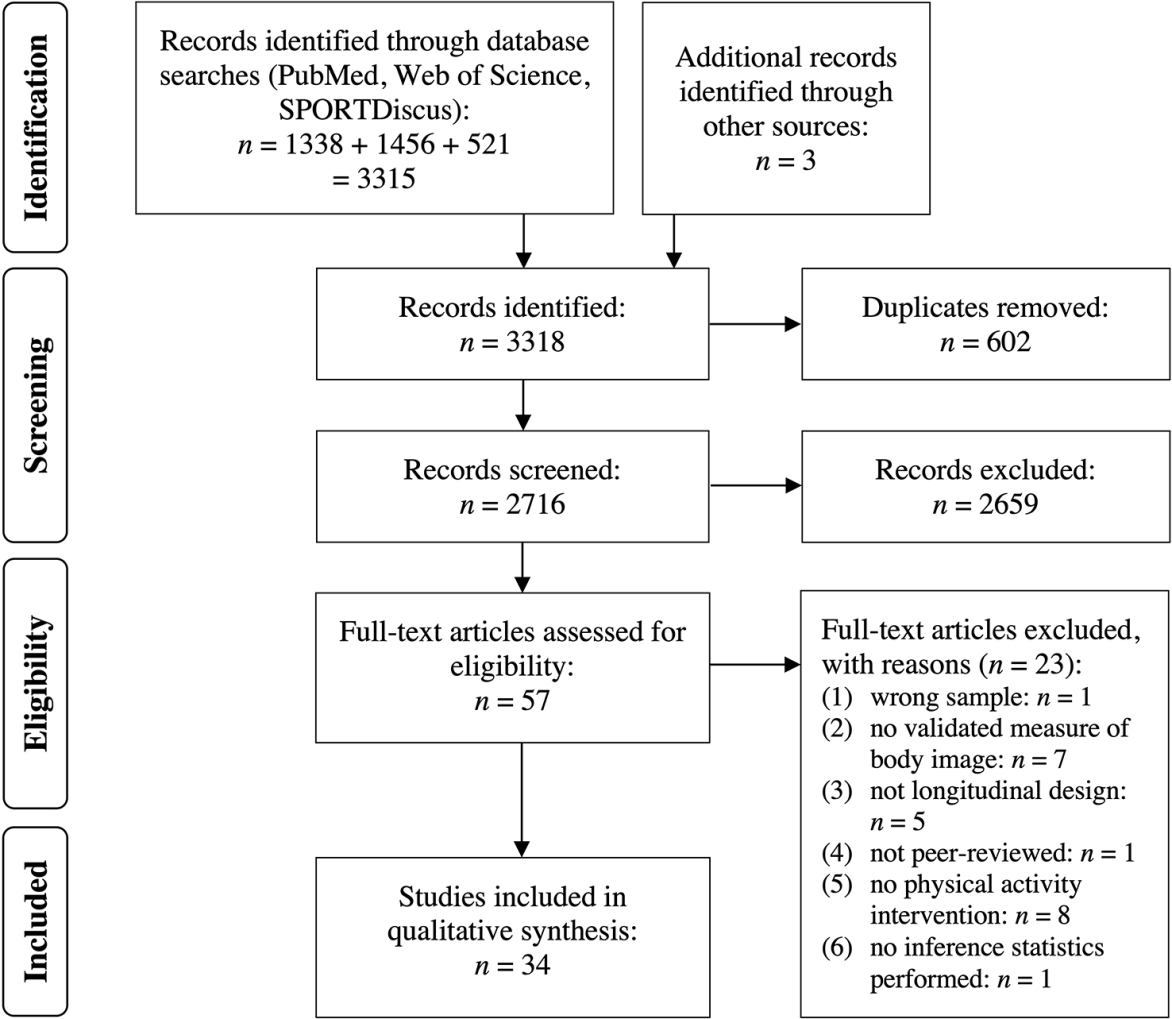
PRISMA flowchart for study selection.

The searches yielded a total of 3,318 results. Duplicates were discarded (n = 602), leaving 2,716 records for title and abstract screening. During this process, 2,659 studies were discarded, and the remaining 57 studies were identified for full-text analysis. Subsequently, 34 studies were included in the systematic review. For the PRISMA flow chart, see **Figure 1**.

### Study Quality

The overall study quality within the current review showed a slight majority for “weak” (55.88%; n = 19), followed by “moderate” (41.17%; n = 14). Only 1 study (2.94%) included in the review qualified for the global “strong” rating. The detailed components ratings are included in **Table 1**.

**Table 1:** Overview on main characteristics and findings of included studies.

Study	Sample characteristics	*n*	Physical activity intervention & dosage	Measure of fitness level	Measure of body composition	Measure of body image/representation	Main findings	Quality rating: Strong 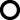 Moderate 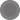 Weak 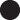						
								Global rating	Selection bias	Study design	Confounders	Blinding	Data collection methods	Withdrawal and dropouts
Folkins (32) 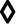	M_age_: *n. r*.	18	Unspecified 36x *n. r*. minutes over 12 weeks	VO_2_max	N/A	BCS	**PI & Control Groups** **•** There were no significant changes in BCS scores post-test for both groups.	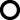	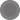	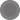	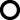	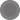	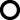	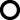
	M_BMI_: *n. r*. % Female: 0% ※At high-risk of coronary heart disease **Controls:**	18												
	no intervention													

Tucker (33) 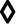	M_age_: *n. r*.	60	Weight training 32x 50 minutes over 16 weeks	N/A	N/A	TSCS	**PI Group** **•** Showed significant increase from pre to post PI on all TSCS indices except Social Self. **•** Scored significantly higher than control group in the Total Positive, Identity, Behaviour, Physical Self, and Personal Self indices of TSCS post PI. **Control Group** **•** There were no significant changes in TSCS scores post-test. ※PI and control groups did not differ significantly in Moral-Ethical Self, Self-Satisfaction, Family Self, or Social Self indices post-test.	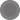	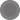	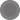	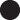	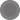	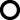	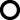
	M_BMI_: *n. r*. % Female: 0% **Controls:**	45												
	no intervention													
Tucker (34) 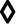	M_age_: *n. r*. M_BMI_: *n. r*. % Female: 0% **Controls:** no intervention	142 130	Strength (weight) training 32x 50 minutes over 16 weeks	Strength test	N/A	BCS, TSCS	**PI Group** **•** Scored significantly higher than control group for both BCS and TSCS measures post PI. **Control Group** **•** There were no significant changes in BCS and TSCS scores post-test.	** 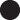 **	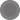	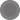	** 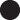 **	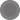	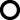	** 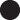 **

Tucker (35) 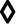	M_age_: *n. r*. M_BMI_: *n. r*. % Female: 0% **Controls:** no intervention	114 127	Weight training 32x *n. r*. minutes over 16 weeks	1-RM strength test		BCS	**PI Group** **•** Scored significantly higher than control group for BCS post PI. **Control Group** **•** There were no significant changes in BCS scores post-test.	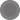	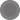	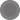	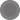	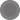	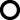	** 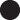 **

Caruso and Gill (36) 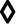	Study 1 M_age_: *n. r*. M_BMI_: *n. r*. % Female: 100% **Controls:** physical education activity class Study 2 M_age_: *n. r*. M_BMI_: *n. r*. % Female: 45.2% **Controls:** non-fitness activity	13 15 6 42 23	Weight training Aerobic training 30x 50 minutes over 10 weeks Weight training 30x 50 minutes over 10 weeks	1-RM strength test, VO_2_max Max. repetition strength test	Body fat % (skinfold measures at 3 sites) Body fat % (skinfold measures at 3 sites)	PSPP, PIP, BCS PSPP, PIP, BCS, BES, Stunkard Scale of Silhouette	**PI & Control Groups** **•** PSPP, PIP and BCS scores improved post-test (significance not calculated) but did not significantly differ between groups. **PI & Control Groups** **•** PSPP, PIP, BCS, BES scores and Body Size Drawings revealed no significant changes post-test for both groups.	** 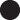 **	** 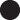 **	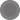	** 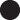 **	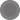	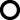	** 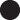 **

Tucker and Maxwell (37) 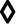	M_age_: *n. r*. M_BMI_: *n. r*. % Female: 100% **Controls:** exercised 2.9 ± 2.2 days/week; no weight training	60 92	Weight training 30x 45 minutes over 15 weeks	1-RM strength test	Body fat % (skinfold measures at 3 sites)	BCS	**PI Group** **•** Scored significantly higher than control group for BCS post PI (after controlling for pre-test differences). **•** Showed significantly greater improvement than control group from pre-test to post-test on BCS scores. **Control Group** **•** Showed no significant improvement from pre-test to post-test on BCS scores.	** 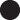 **	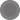	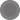	** 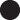 **	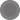	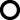	** 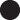 **

Tucker and Mortell (38) 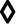	M_age_: 42.5 ± 4.2 M_BMI_: *n. r*. % Female: 100% **Controls:** none	30 30	Weight training Walking program 36x *n. r*. minutes over 12 weeks	Max. repetition & 1-RM strength test, 1 Mile Walk test	N/A	BCS	**PI Groups** **•** Both groups showed significant improvement in BCS scores post PI. **•** Weight-trainers scored significantly higher than walkers on BCS scores post PI.	** 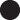 **	** 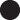 **	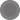	** 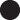 **	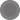	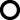	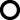

McAuley et al. (39) 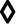	M_age_: 54.5 M_BMI_: *n. r*. % Female: 50.6% **Controls:** none	83	Aerobic training 60x 40 minutes over 20 weeks	VO_2_max	Body fat % (skinfold measures at 3 sites)	PSPP, PIP	**PI Group** **•** Significant improvements were found in the following subscales of PSPP: Physical Self-Worth & perceptions of Physical Condition. **•** Attractive Body subscale of PSPP rating showed no significant effect post-test.	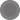	** 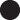 **	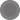	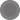	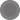	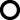	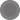

McAuley et al. (40) 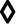	M_age_: 66.71 ± 5.35 M_BMI_: *n. r*. % Female: 71.8% **Controls:** none	85 89	Aerobic training Toning exercise 72x 40 minutes over 24 weeks	VO_2_max, heartrate	Body fat % (TOBEC)	PSPP	**PI Groups** **•** Latent growth curve analyses showed a curvilinear pattern of growth with significant increases at all levels of PSPP measure upon completion of PI. **•** Significant declines were shown at 6 months post PI in both groups.	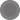	** 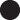 **	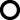	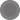	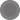	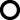	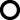

Williams and Cash (41) 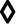	M_age_: 21.7 ± 3.8 M_BMI_: 23.9 ± 4.4 % Female: 69.2% **Controls:** no intervention	39 39	Weight training 1x 180 minutes over 6 weeks	1-RM strength test	N/A	MBSRQ, SPAS	**PI Group** **•** Showed significant improvement post PI in SPAS scores, as well as Appearance Evaluation and Body Area Satisfaction subscales of MBSRQ. **Control Group** **•** Reported no significant changes on all measures post-test. ※After adjusting for pre-test score differences, significant group differences were found for Appearance Evaluation, Body Area Satisfaction and Social Physique Anxiety post-test.	** 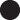 **	** 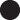 **	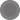	** 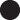 **	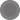	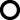	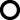

Aşçı (42) 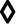	M_age_: 22.15 ± 1.87 M_BMI_: *n. r*. % Female: 52.9% **Controls:** no intervention	70 68	Step dance 30x 50 minutes over 10 weeks	N/A	N/A	PSPP	**PI & Control Groups** **•** PI group showed significantly greater improvement than control group from pre-test to post-test on all PSPP subscales except for Sport Competence.	** 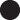 **	** 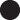 **	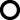	** 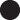 **	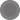	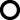	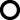

Aşçı (43) 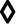	M_age_: 21.35 ± 0.88 M_BMI_: *n. r*. % Female: 100% **Controls:** no intervention	20 20	Aerobic training & step dance 30x 50 minutes over 10 weeks	N/A	N/A	PSDQ	**PI & Control Groups** **•** Participants in the PI group showed significant improvement in Physical Activity, Coordination, Sport Competence and Flexibility subscales of PSDQ as compared the control group post-test.	** 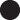 **	** 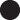 **	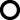	** 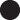 **	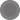	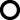	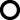
Depcik and Williams (44) 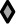	M_age_: 22.13 ± 5.51 M_BMI_: *n. r*. % Female: 100% ※Pre-existing body image disturbance (109.0 mean group score on BSQ) **Controls:** exercised 2.23 ± 2.02 days/week; no weight training	15 15	Resistance (weight) training 52x 50 minutes over 13 weeks	1-RM strength test	N/A	BCS, BSQ	**PI & Control Groups** **•** Mean BCS scores significantly increased for weight trainers as compared to control group post-test. **•** The groups differed significantly in body image disturbance (BSQ scores) post-test. Weight trainers experienced a greater reduction in body image disturbance than control group post-test.	** 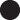 **	** 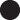 **	** 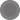 **	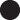	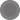	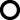	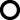

Annesi (45) 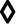	M_age_: *n. r*. M_BMI_: *n. r*. % Female: 100% **Controls:** no intervention	48 30	Cardiovascular training 36x 30 minutes over 12 weeks	N/A	N/A	BES	**PI & Control Groups** **•** Scores on the Physical Condition subscale of BES significantly increased post-test for the exercise group, but not the control group. **•** Scores on the Sexual Attractiveness subscale of BES did not significantly change post-test for both groups.	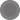	** 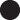 **	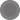	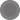	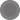	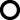	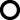

Annesi and Westcott (46) 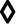	M_age_: 46.3 ± 13.4 M_BMI_: *n. r*. % Female: 50.6% **Controls:** none	35	Weight & cardiovascular training 30x 60 minutes over 10 weeks	Heartrate	N/A	PSPS	**PI Group** **•** PSPS scores significantly improved post PI.	** 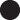 **	** 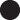 **	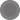	** 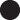 **	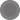	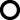	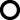

Ginis et al. (47) 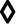	M_age_: 21.57 ± 2.47 M_BMI_: *n. r*. % Female: 36.4% **Controls:** none	44	Strength (weight) training 60x *n. r*. minutes over 12 weeks	1-RM test	Body fat % (DXA)	SPAS, BASS, DMS	**PI Group** **•** Participants experienced significant increases in BASS and decreases in SPAS scores post PI. **•** Only male participants experienced a significant decrease in muscularity dissatisfaction (DMA score) post PI.	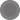	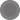	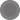	** 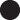 **	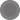	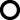	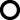

Hős (48) 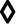	M_age_: 48.9 ± 5.6 M_BMI_: *n. r*. % Female: 100% **Controls:** no intervention	25 28	Aerobic dance 52x 60 minutes over 52 weeks	N/A	N/A	TSIT	**PI Group** **•** Showed significant increases post PI on Total Self-Image (all subscales of TSIT, except for Family Self-Image). **Control Group** **•** There were no significant changes post-test on Total Self-Image (all subscales of TSIT, except for Social Self-Image). ※There were significant differences between both groups on all subscales of TSIT post-test, except for Social Self-Image.	** 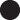 **	** 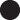 **	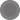	** 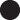 **	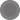	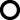	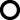

Henry et al. (49) 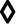	M_age_: 19.24 ± 1.43 M_BMI_: 22.85 ± 3.39 % Female: 100% **Controls:** low to moderate exercise (3.24 days/week)	23 28 21	Aerobic training Interval cuircuit (weight) training 36x 50 minutes over 12 weeks	Step test (VO_2_max), bench press test (muscular strength & endurance)	Body fat % (skinfold measure at 3 sites)	BSIQ	**PI & Control Groups** **•** Interval circuit training group improved significantly post PI in overall Appearance Evaluation and Health/Fitness Evaluation & Influence, as well as significantly reduced Negative Affect subscales of the BSIQ as compared to control group.	** 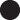 **	** 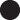 **	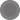	** 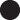 **	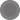	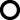	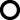

Opdenacker et al. (50) 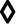	M_age_: 66.65 ± 4.16 M_BMI_: 27.08 ± 19.11 % Female: *n. r*. **Controls:** no intervention	46 49 46	Lifestyle PI Structured exercise 33x 60-90 minutes over 11 weeks	VO_2_max	N/A	PSPP	**PI & Control Groups** **•** Immediately after PI, the lifestyle group showed significant improvements in Self-Perceived Physical Condition, Body Attractiveness, and Physical Self-Worth subscales of PSPP. In the structured group, significant effects were found on only on Physical Condition. **•** One year after PI, the lifestyle group had significant effects on Body Attractiveness while the structured group showed significant improvements in Physical Condition and Body Attractiveness. ※There were no significant differences between PI groups for both short and long-term results.	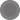	** 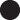 **	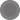	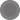	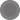	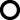	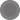

Özdemir et al. (51) 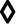	M_age_: *n. r*. M_BMI_: *n. r*. % Female: 0% **Controls:** no intervention	11 11 11 12	Swimming Cycling Running 36x 40 minutes over 12 weeks	VO_2_max	Body fat % (DEXA), muscular strength (isokinetic dynamometer),	PSPP	**PI & Control Groups** **•** All groups revealed no statistical improvement in PSPP scores post-test.	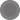	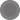	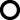	** 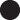 **	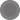	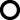	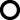

Cruz-Ferreira et al. (52) 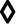	M_age_: 41.08 ± 6.64 M_BMI_: *n. r*. % Female: 100% **Controls:** no intervention	38 24	Pilates 48x 60 minutes over 24 weeks	N/A	N/A	PSCS	**PI Group** **•** Showed significant improvement between baseline and 6 months post PI in Perception of Physical Appearance, Functionality and Total Physical Self-Concept. **Control Group** **•** No significant differences were observed over time. ※No significant differences in PSCS scores were observed between groups at all time points.	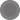	** 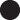 **	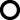	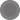	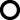	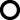	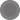

Moore et al. (53) 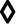	M_age_: 20.2 ± 2.02 M_BMI_: *n. r*. % Female: 30.8% **Controls:** none	120	Resistance (weight) training 24x *n. r*. minutes over 12 weeks	1-RM strength test	N/A	PSPS, PSAQ	**PI Group** **•** Significant improvements were observed across all measures post PI.	** 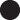 **	** 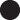 **	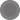	** 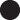 **	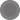	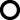	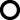

Van Puymbroeck et al. (54) 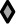	M_age_: 58.67 ± 9.82 M_BMI_: *n. r*. % Female: 100% ※≥9 months post breast cancer treatment **Controls:** light exercise	18 12	Hatha yoga 16x 75 minutes over 8 weeks	Tests for flexibility, strength, abdominal muscular endurance, agility & dynamic balance	N/A	Body Image Scale for use with cancer patients	**PI & Control Groups** **•** PI group reported significantly more positive body image as compared to control group at pre and post-test. **•** There were no statistical differences for the changes in body image scores over time for both groups.	** 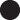 **	** 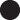 **	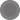	** 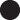 **	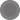	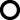	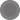

Appleton (55) 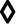	M_age_: *n. r*. M_BMI_: *n. r*. % Female: 52.9% **Controls:** within-subjects, no intervention (12x 40 minutes reading over 2 weeks)	34 34	Cardiovascular training 12x 40 minutes over 2 weeks	N/A	Body weight, waist & hip circumferences	MBSRQ	**PI Group** **•** There were significant increases in the following subscales of MBSRQ: Appearance Evaluation, Fitness Evaluation, Fitness Orientation, Health Evaluation, Illness Orientation and Body Areas Satisfaction; Self-Classified Weight significantly decreased post PI. **Control Group** **•** Appearance Evaluation and Body Areas Satisfaction subscales of MBSRQ significantly decreased during no PI condition. ※Appearance Orientation, Health Orientation and Overweight Preoccupation subscales of MBSRQ were unaffected by PI.	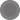	** 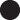 **	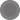	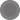	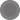	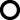	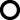

Hatipoglu et al. (56) 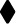	M_age_: 45.63 ± 8.12 M_BMI_: 33.2 % Female: 18.2% ※Acromegaly patients **Controls:** no intervention	11 9	Cardiovascular, strength, balance & stretching training 36x 75 minutes over 12 weeks	N/A	BMI	MBSRQ	**PI Group** **•** Significant improvement in MBSRQ scores was observed post PI. **Control Group** **•** No significant changes in MBSRQ scores were reported post-test.	** 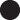 **	** 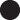 **	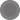	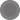	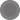	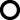	** 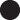 **

Pearson and Hall (57) 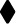	M_age_: 33.4 ± 7.6 M_BMI_: 29.02 ± 4.71 % Female: 100% ※Obesity patients (BMI >25 kg/m^2^) **Controls:** none	37	Cardiovascular training 54x 45 minutes over 18 weeks	VO_2_max	Waist circumference, body weight & fat % (DXA)	MBSRQ	**PI Group** **•** Significant improvements occurred between baseline and week 6 as well as week 18 post PI for Appearance Evaluation, Fitness Orientation and Body Areas Satisfaction subscales of MBSRQ.	** 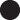 **	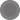	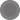	** 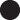 **	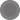	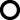	** 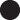 **

Seguin et al. (58) 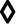	M_age_: 62 ± 12 M_BMI_: *n. r*. % Female: 100% **Controls:** none	341	Strength (weight) training 20x 60 minutes over 10 weeks	N/A	N/A	MBSRQ	**PI Group** **•** Significant improvement occurred post PI for Health Orientation, Subjective Weight, Fitness Orientation, Fitness Evaluation, and Health Evaluation subscales of MBSRQ. **•** There were no significant changes in Weight Preoccupation subscale of MBSRQ post PI.	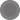	** 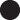 **	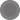	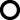	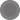	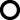	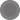

Zarshenas et al. (59) 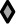	M_age_: 26 ± 6.9 M_BMI_: *n. r*. % Female: 100% ※Pre-exisiting self-reported mild to severe depressive symptoms (BDI-II score of ≥14) **Controls:** no intervention	41 41	Aerobic training *n. r*.x 65 minutes over 4 weeks	Heartrate	N/A	MBSRQ	**PI Group** **•** MBSRQ scores across all subscales significantly improved post PI. **•** Significant improvement was found in Appearance Evaluation, Appearance Orientation, Health Orientation, and Illness Orientation subscales of MBSRQ post PI as compared to control group. **Control Group** **•** There were no significant changes in MBSRQ scores post-test.	** 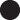 **	** 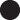 **	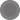	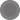	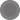	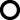	** 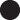 **

Ginis et al. (60) 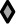	M_age_: 21.5 ± 2.93 M_BMI_: 22.96 ± 3.89 % Female: 100% ※Pre-existing body image concerns (≥27 score on SPAS & ≤3 on BASS) **Controls:** none	17 23	Aerobic training Strength training 24x 45 minutes over 8 weeks	VO_2_max, 10-RM strength test	BMI, waist-hip ratio	SPAS, AE, BASS, PSDQ	**PI Groups** **•** Both PI groups revealed significant improvements across all measures post PI. **•** Aerobic training group yielded significantly greater improvements in SPAS as compared to strength training group post PI.	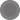	** 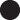 **	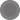	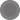	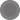	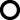	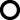

Mendonça et al. (61) 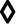	M_age_: *n. r*. M_BMI_: *n. r*. % Female: 100% **Controls:** no intervention	25 28 21 25	Strength (weight) training Dance Hydrogymnastics 48x 60 minutes over 16 weeks	8-RM strength test, heart rate	N/A	SPA, Stunkard Scale of Silhouette	**PI and Control Groups** **•** Significant improvements in SPA scores were found regardless of the program, with the greatest effect shown by the strength training group post PI. **•** No significant differences were found for body image perception and bodily dissatisfaction post PI. **•** All PI groups showed significant improvements across all measures when as compared to control group post-test.	** 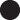 **	** 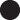 **	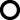	** 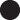 **	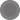	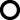	** 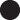 **

Vurgun (62) 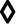	M_age_: 40.5 ± 12.1 M_BMI_: *n. r*. % Female: 100% **Controls:** no intervention	12 20	Aerobic training 42x 60 minutes over 14 weeks	Heartrate	BMI, waist-hip ratio, body density and fat ratio (skinfold measured at 9 sites)	BISQ	**PI and Control Groups** **•** BISQ scores were significantly improved post PI as compared to control group.	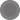	** 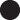 **	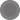	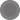	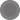	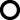	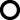

Baur et al. (63) 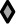	M_age_: 37.9 ± 9.2 M_BMI_: *n. r*. % Female: 52.8% ※Suffers from non-specific back pain **Controls:** none	17	Fascial* fitness 3x 60 minutes over 3 weeks	N/A	N/A	FKB-20	**PI groups** **•** Fascial fitness group showed significant improvement only for negative body image post PI.	** 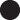 **	** 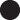 **	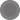	** 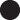 **	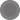	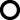	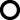

Megakli et al. (64) 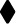	M_age_: 32.70 ± 7.26 M_BMI_: 35.84 ± 4.59 % Female: 100% ※ Obesity patients (BMI >30 kg/m^2^) **Controls:** no intervention	18 19	Aerobic & resistance training 36x 30 minutes over 12 weeks	N/A	Waist & hip circumferences	PSPP	**PI and Control Groups** **•** PI group showed significant increases post PI for all PSPP subscale scores except for Perceived Body Attractiveness as compared to control group.	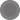	** 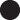 **	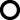	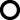	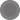	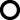	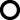
Aukštuolytė et al. (65) 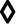	M_age_: 35.58 M_BMI_: *n. r*. % Female: 100% **Controls:** none	15 16	Functional training Zumba 16x 60 minutes over 8 weeks	N/A	Body fat % (skinfold measure at 4 sites)	BSQ, Figure Rating Scale	**PI Groups** **•** Body shape dissatisfaction was significantly reduced for functional training group after PI, but not for Zumba group.	** 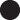 **	** 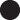 **	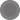	** 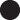 **	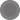	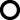	** 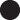 **

**Symbols**: [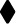] Clinical sample; [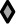] Sub-clinical sample; [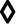] Non-clinical sample.

**Abbreviations**: Not Applicable (N/A); Not Reported (n.r.); Physical Activity Intervention (PI).

**Abbreviated Measures**: Appearance Evaluation (AE) sub-scale of MBSRQ; Beck's Depression Inventory-second edition (BDI-II); Body Areas Satisfaction (BASS) sub-scale of MBSRQ; Body Cathexis Scale (BCS); Body Esteem Scale (BES); Body Image Satisfaction Questionnaire (BISQ); Body Self-Image Questionnaire (BSIQ); Body Shape Questionnaire (BSQ); Drive for Muscularity Scale (DMS); Fragebogen zum Körperbild (FBK-20 aka. Body Image Questionnaire (BIQ)); Multidimensional Body Self-Relations Questionnaire (MBSRQ); Perceived Importance Profile (PIP); Physical Self-Attribute Questionnaire (PSAQ); Physical Self-Concept Scale (PSCS) subscale of the TSCS; Physical Self-Description Questionnaire (PSDQ); Physical Self; Perception Profile (PSPP); Physical Self-Perception Scale (PSPS) subscale of the TSCS; Satisfaction with Physical Appearance Scale (SPA); Social Physique Anxiety Scale (SPAS); Tennessee Self Concept Scale (TSCS); Tennessee Self Image Test (TSIT).

*The Structural Integration group from (63) is not taken into consideration, as structural integration is not considered as a physical activity intervention, rather a form of physical therapy.

Notably, included studies only received weak (n = 26) and moderate (n = 8) ratings for the selection bias category. The main reason for depreciation was the recruitment in sports classes or from community samples; thus producing a selection bias favouring highly motivated, sports-oriented participants. In the same vein, most studies qualified for moderate rating in the study design category (n = 27). This is due to the lack of randomisation in the selection as well as group allocation process, as participants often conducted the PI of their choice. This limits explanatory power regarding general recommendations of effective types of training. To summarise, there is a possibility for risk of bias in the studies included in the current review.

### Study Characteristics

The study characteristics of included studies are detailed in **Table 1**. All studies employed a longitudinal design, as defined by the inclusion criteria. Of the total 34, 6 studies were described as quasi-experimental. Twenty-three studies included control groups in their experimental design, while the remaining 11 did not. Within the 23 controlled studies, 18 studies included “no intervention” comparison groups, while the remaining 5 studies included participants who performed low to moderate exercise as controls. Eight studies included clinical and/or sub-clinical groups. The remaining 26 studies had healthy samples. Eleven studies aimed to compare different types of PIs and their impact on body image/body representation of participants. Most studies (n = 27) did not have a randomised participant selection and/or group allocation process.

### Physical Activity Interventions

The most commonly investigated types of PI were weight/strength training (n = 14), as well as aerobics/cardiovascular training (n = 13). Three studies implemented the combination of both types as a singular intervention. Dancing (n = 3) was also investigated. Other PIs included walking, running, swimming, cycling, pilates, hydrogymnastics, yoga, fascial fitness, and functional training. The mean length of time for the implementation of the PI was 13.29 (SD = 8.45) weeks, with the maximum of 52 weeks (1 year) and the minimum of 2 weeks. The median for PI duration was 12 weeks.

VO_2_max was used as a measure of fitness in 9 studies. Eleven studies conducted strength tests as a marker of fitness level (i.e., variations of maximum repetition test). Heartrate was also measured as a marker of fitness level (n = 4). Twelve studies did not report any measure of physical fitness.

### Outcome Measures

Studies were homogeneous in terms of the operationalisation of body image/body representation measures. Almost all studies exclusively implemented validated questionnaires assessing body image. Only 3 studies additionally implemented more visual-oriented measures (i.e., Stunkard Scale of Silhouette, Figure Rating Scale)—though nevertheless still affective/subjective in nature. No study used experimental assessments of body representation (e.g., depictive/metric body size and visual estimation tasks). As such, the domains of visual, tactile and affordance perception of body representation were not at all investigated.

The most commonly employed scales were MBSRQ (Multidimensional Body Self-Relations Questionnaire; (66) and PSPP (Physical Self-Perception Profile; (67) at 23.53% (n = 8) each. The Body Cathexis Scale was also frequently implemented in earlier studies (n = 7; 20.59%; (68).

#### Systematic Effects of PI on Body Representation

PI was considered effective if significant improvement in body representation measures was reported at post-test among the intervention group, relative to the control group. The overall results are as follows: 5 studies (14.71%) observed no significant improvement in both control and PI groups across all body image measures; 3 studies (8.82%) observed significant improvement in both control and PI groups across all body image measures; 10 studies (29.41%) reported significant improvement in PI group across all body image measures; 16 studies (47.06%) reported partial significance effect in PI groups (i.e., not all improvement in scores measured in the subscales of the implemented questionnaires reached significance).

However, due to the low number of clinical and sub-clinical studies (n = 8) included in this systematic review, we cannot reliably synthesise significant evidence with regard to utilising long-term PI (structured or otherwise) as a treatment measure for clinically relevant groups.

#### Mechanisms Underpinning the Interplay Between PI and Body Representation

All studies included neither explicitly addressed/proposed a form of a mechanistic interplay between PI and body representation, nor provided evidence to support one. As such, the question of which potential mechanisms might be responsible for the apparent shift in body representation after the introduction of physical activity remain inadequately addressed.

## Discussion

In this systematic review, we synthesised the empirical findings from longitudinal intervention studies on effects of structured PI on body representation. Overall, the studies suggest that the implementation of structured PI is associated with improved body image. Effects on other body representations were not investigated. To our knowledge, this review is the first to demonstrate such an effect in longitudinal settings. The effectiveness of these interventions seems promising for future research and possible development of prevention interventions. However, we argue that due to the quality of existing studies, further research is highly necessary to investigate whether the positive effects of PI can be generalised across healthy (active or sedentary), sub-clinical as well as clinical populations, and to capture a more complete sense of body representations that allows for exploring potential mechanisms of change.

Based on the evidence available, PI can be cautiously regarded as a potentially effective option regarding body image related outcomes. Notably, the studies included in the review examined predominantly volunteer groups (i.e., people who were generally open to the idea of engaging in physical activity), even if they were previously sedentary. It remains unclear whether positive effects of PI would also generalise to the voluntarily sedentary population who are physical-activity-reluctant. From a clinical perspective, these groups would be very relevant, since they are at higher risk for cardiovascular diseases and obesity. Thus, from the current studies, it remains unclear whether PI can be recommended as a general treatment to people suffering from poor body image.

Based on our systematic review, it would not be responsible to simply conclude that PI might be a promising treatment option for sub-clinical and/or clinical populations whose core psychopathology is centred around body dissatisfaction and distorted representation of one's own body. Despite the fact that a general improvement in measures of body representations can be observed, the percentage of clinical and sub-clinical studies included within the systematic review is too low to draw such a conclusion (23.53%). Additionally, there is no clear evidence regarding an additional or interactive effect of PI when implemented in conjunction with established treatments for clinical populations. More importantly, a previous meta-analytic review of stand-alone interventions to improve body image by Alleva and colleague (18) has provided evidence which cautioned against discussing physical activity with patients, as it was significantly associated with poorer body image outcomes. The meta-analysis proposed that by discussing physical activity as an intervention, patients may inadvertently have their attention drawn to their own weight and appearance, as well as the associated societal standards for physical fitness and physical attractiveness. Further, it was also not reported to be significantly associated with larger intervention effects on body image. Until the literature on the underlying mechanism between physical activity and body representation is further investigated, physical activity-related interventions targeting body image/representation should therefore be exclusively kept to psychologically healthy populations or be closely embedded in an overall treatment concept.

Notably, objective improvements in bodily composition and physical fitness brought about by PI are inconsistently related to changes in body image. This is surprising, insofar as people typically assume that their body image is based on an objective evaluation and comparison of their body. Instead, it appears that complex appraisal processes, eventually involving perceived improvements in physical capacities or more intense somatosensation experiences during PI may play a more important role. PI interventions could serve to improve body image/body representation by allowing individuals to redirect their attention more toward the functionality of their body and less on their appearance, or by increasing their sense of physical efficacy (69, 70). In this sense, the previous literature supports the need for a comprehensive, multisensory assessment of body representation as suggested by the Longo framework.

### Strengths and Methodological Considerations

To our knowledge, this review is the first to provide a comprehensive systematic review on the topic of the longitudinal interactions between PI and body representation—the definition of which we have updated and adapted to fit the more complex theories and discussions which have arisen over the years.

Methodological limitations of this review arise from our study selection process as well as fromthe included studies. As we only searched for published results, a publication bias towardsignificant effects cannot be excluded. Further, as terminology in the field is very heterogeneous,it is possible that despite our broad search strategy, a few relevant articles may have been missed.Notably, some of the included studies had small sample sizes and may have been underpowered. Thecurrent systematic review is also potentially limited by biases within studies. Although nosystematic risk of bias across or within studies were identified, 97% of the included studies wereconsidered at risk with regards to selection bias and study design. More importantly, all studiesare lacking in the variety of validated outcome measures. Only self-report questionnaires wereimplemented, and the main component of body image addressed here was body satisfaction or the lackthereof. Additional visual scales implemented were used to measure the disparity between participants’ subjective ideal versus actual body shapes, which, once again, only measured participants’ attitudinal/conceptual issues of their own body image. Moreover, the two studies whose results also reported long-term effects of PI on body image were shown to be in direct contradiction (40, 50). One possible explanation for the contrasting results might be the difference in the type and dosage of the PIs implemented. As such, it remains unclear whether PI-induced body image improvement is indeed sustainable.

### Perspectives and Future Directions

Our systematic review revealed that evidence on PI as a means to change body representation is still limited. A major challenge for future research is not only to reduce selection bias in the investigated samples, but also to explore potential mechanisms of body image improvement *via* PI through adopting a broader perspective on body representation. Based on our review, we argue for a more comprehensive view that takes various sources of information about the body into account (71, 72). In pursuit of a mechanism-oriented intervention, it is imperative to have a solid grasp on the understanding of how body image/body representation are constructed and which aspects drive changes in how individuals mentally represent their body.

The assessment of multisensory body representation is challenging. However, an increasing number of experimental paradigms have been developed in recent years to assess such concepts as: interoception [e.g., (73–75)], implicit knowledge of body dimensions (76–78) and multisensory integration (24). Despite reports of potentially disturbed multisensory integration and interoception in eating disorders (24, 79, 80), these measures have so far been largely neglected in clinical research. We expect that a broader use and further development of these methods in body representation assessment could give rise to a more informed understanding of the mechanisms of disturbed body representation and its malleability.

To this end, it is important to undertake future research on (i) identifying valid tasks to investigate different body representations (e.g., through combining actual body measures with tasks assessing body size estimation, interoceptive abilities or affordance estimates with questionnaires assessing cognitive-affective appraisal of the body), and (ii) investigate the malleability and interactions between different body representations.

## Author Contributions

DS, KG, SB, SZ, and AT conceptualized the project. DS, SB, and L-MW performed the literature review. DS wrote the first draft of the manuscript. SB, KG, HW, and AT critically reviewed the manuscript with respect to their areas of expertise: body image and eating disorders (KG, SB, SZ); philosophy and cognitive science of body perception (HW); and sports science (AT).

## Funding

This review was supported by a grant from the intramural graduate school “iReAct” of the University and the University Hospital Tübingen. We acknowledge support by Open Access Publishing Fund of the University Tübingen.

## Conflict of Interest

The authors declare that the research was conducted in the absence of any commercial or financial relationships that could be construed as a potential conflict of interest.
